# Neurological manifestations of nontuberculous mycobacteria in adults: case series and review of the literature

**DOI:** 10.3389/fneur.2024.1360128

**Published:** 2024-04-26

**Authors:** Yair Mina, Ahnika Kline, Maura Manion, Dima A. Hammoud, Tianxia Wu, Julie Hogan, Irini Sereti, Bryan R. Smith, Christa S. Zerbe, Steven M. Holland, Avindra Nath

**Affiliations:** ^1^National Institute of Neurological Disorders and Stroke, National Institutes of Health, Bethesda, MD, United States; ^2^Sackler Faculty of Medicine, Tel-Aviv University, Tel-Aviv, Israel; ^3^National Institute of Allergy and Infectious Diseases, National Institutes of Health, Bethesda, MD, United States; ^4^Center for Infectious Disease Imaging, Radiology and Imaging Sciences, Clinical Center, National Institutes of Health, Bethesda, MD, United States

**Keywords:** infections, neuroinfectious diseases, nontuberculous mycobacteria, magnetic resonance imaging, neuroimaging

## Abstract

**Introduction:**

Nontuberculous mycobacteria (NTM) mediated infections are important to consider in cases with neuroinflammatory presentations. We aimed to characterize cases of NTM with neurological manifestations at the National Institutes of Health (NIH) Clinical Center and review the relevant literature.

**Materials and methods:**

Between January 1995 and December 2020, six cases were identified. Records were reviewed for demographic, clinical, and radiological characteristics. A MEDLINE search found previously reported cases. Data were extracted, followed by statistical analysis to compare two groups [cases with slow-growing mycobacteria (SGM) vs. those with rapidly growing mycobacteria (RGM)] and evaluate for predictors of survival. NIH cases were evaluated for clinical and radiological characteristics. Cases from the literature were reviewed to determine the differences between SGM and RGM cases and to identify predictors of survival.

**Results:**

Six cases from NIH were identified (age 41 ± 13, 83% male). Five cases were caused by SGM [*Mycobacterium avium* complex (MAC) *n* = 4; *Mycobacterium haemophilum n* = 1] and one due to RGM (*Mycobacterium abscessus*). Underlying immune disorders were identified only in the SGM cases [genetic (*n* = 2), HIV (*n* = 1), sarcoidosis (*n* = 1), and anti-interferon-gamma antibodies (*n* = 1)]. All cases were diagnosed using tissue analysis. A literature review found 81 reports on 125 cases (SGM *n* = 85, RGM *n* = 38, non-identified *n* = 2). No immune disorder was reported in 26 cases (21%). Within SGM cases, the most common underlying disease was HIV infection (*n* = 55, 65%), and seizures and focal lesions were more common. In RGM cases, the most common underlying condition was neurosurgical intervention or implants (55%), and headaches and meningeal signs were common. Tissue-based diagnosis was used more for SGM than RGM (39% vs. 13%, *p* = 0.04). Survival rates were similar in both groups (48% SGM and 55% in RGM). Factors associated with better survival were a solitary CNS lesion (OR 5.9, *p* = 0.01) and a diagnosis made by CSF sampling only (OR 9.9, *p* = 0.04).

**Discussion:**

NTM infections cause diverse neurological manifestations, with some distinctions between SGM and RGM infections. Tissue sampling may be necessary to establish the diagnosis, and an effort should be made to identify an underlying immune disorder.

## 1 Introduction

Nontuberculous mycobacteria (NTM) comprise mycobacterial species other than *Mycobacterium tuberculosis* and *Mycobacterium leprae*. It is an important group of human pathogens that predominantly affect immunocompromised hosts (1). It includes two major groups defined by their ability to grow in solid culture media: (a) slow-growing mycobacteria [SGM, e.g., *Mycobacterium avium* complex (MAC)] causing mainly pulmonary or disseminated disease, and (b) rapidly growing mycobacteria (RGM, e.g., *Mycobacterium abscessus*) which mainly cause skin, soft tissue, pulmonary, or disseminated infection. Both groups have been shown to also cause neurological disease (2, 3).

The most common neurological manifestation of NTM is meningitis (4), but there are also reports of parenchymal disease due to hematogenous spread in disseminated disease (5) or regional spread from adjacent tissue or infected devices (6). Neurological manifestations are increasingly reported among patients with HIV/AIDS (7) and other immunodeficiencies, as well as patients with histories of neurosurgery and intracranial implants. NTM are challenging to diagnose and treat, with high rates of mortality and long-term morbidity (8).

To better understand and increase clinical awareness of this group of organisms as neurologic pathogens, we report herein the clinical and imaging characteristics of six cases encountered at the NIH in recent years and present an analysis of the relevant literature.

## 2 Materials and methods

### 2.1 NIH cases

A search for cases at the NIH was conducted using two approaches:

1. A search for mycobacterial culture from CSF, brain tissue, or spinal cord tissue which yielded growth of NTM since using the most recent computerized clinical database at NIH (1 August 2004 to 31 December 2020).
2. A search for cases of mycobacterial infection through the available list of patients seen by NIH Neurology consult service (1 January 1995 to 31 December 2020).

Data from cases found using these approaches were reviewed to determine if they were indeed adult NTM cases with neurological manifestations or should be excluded (e.g., *M. tuberculosis* complex infection, age < 16 years, or a case determined clinically to be a result of contamination).

Clinical and ancillary data of patients who were included were obtained by review of charts, focusing on patient characteristics (e.g., any underlying immunodeficiency), clinical events and neurological manifestations, results of ancillary tests including MRI and CSF analysis, treatments, and outcomes.

### 2.2 Standard protocol approvals, registrations, and patient consents

All included cases were seen under a research protocol at the NIH in accordance with the ethical standards as laid down in the 1964 Declaration of Helsinki and its later amendments. Patients were seen under one of two NIH research protocols (NCT00018044; NCT00923065), which were approved by the Institutional Review Board at the NIH. Written informed consent was obtained from all participants.

### 2.3 Literature review

To identify relevant publications with neurological manifestations of NTM, we searched the MEDLINE database, searching the title and abstract for manuscripts with the keywords (“mycobacterium” OR “mycobacteria”) AND (“neuro” OR “meningitis” OR “meningeal” OR “encephalitis” OR “cerebral” OR “nervous” OR “CNS” OR “spine” OR “intracranial” OR “cerebellar” OR “cerebral”). The results were then manually reviewed to identify English-written reports with human adult cases of NTM that described the neurological manifestations and included data on demographics and outcomes. Reported cases were analyzed, with a focus on the initial presentation, underlying conditions, treatment, and clinical evolution. Data were analyzed separately for SGM and RGM to provide descriptive statistics on demographics, medical history, microbiological features, radiological characteristics, treatment regimen, and outcome. In addition, differences between the two groups and between pathogen subgroups within each group were calculated using Fisher’s exact test for categorical variables and one-way analysis of variance for continuous variables. To evaluate for predictors of survival, a multiple logistic regression model was applied using age and sex as covariates. First, a simple logistic regression model was applied to assess the association of survival with each candidate covariate (as listed in Table 1) using a significance level of 0.1. Then, a multiple logistic regression analysis was performed, including all the candidate covariates selected from the first step. The statistical analyses were performed using SAS version 9.4. A significance level (α) of 0.05 was used for all statistical tests.

**Table 1:** Summary of clinical, microbiological, and radiological characteristics of cases of non-tuberculosis mycobacteria with neurological manifestations at the national institutes of health.

Case	Age	Sex	Underlying condition	CD4+ cell count	Pathogen	Neurological manifestations	Imaging findings	Method of diagnosis	Treatment	Outcome
1	40	M	NEMO-deficiency	397	*Mycobacterium avium* complex *(SGM)*	Visual hallucinations, generalized seizure with possible PRES	T2-FLAIR hyperintensities in bilateral occipito-parietal + right hippocampus	CSF culture + culture of brain tissue from autopsy	Azithromycin, meropenem, moxifloxacin, amikacin	Died
2	28	M	NEMO-deficiency	203	*Mycobacterium haemophilum* (SGM)	Progressive quadriparesis	Multiple nodular and ring-enhancing lesions of the brainstem, left frontal lobe and upper cervical cord, progressing to rhomboencephalitis	Acid-fast bacilli stain and PCR from left frontal lesion	Rifabutin, clarithromycin, clofazimine, bedaquiline, moxifloxacin, linezolid, IFN-gamma	Died
3	58	M	HIV	110	*Mycobacterium avium ssp. paratuberculosis* (SGM)	Dysarthria, right central facial nerve palsy, left CN3 palsy, right hemiparesis	Large enhancing lesion in the left midbrain with additional multiple small enhancing lesions in the left frontal lobe and deep gray matter	Positive Fite+AR stains and PCR of tissue from left midbrain biopsy	Rifabutin, ethambutol, azithromycin, moxifloxacin, meropenem, dexamethasone	Resolution
4	56	M	Epidural steroid injection	N/A	*Mycobacterium abscessus* (RGM)	Mild right leg weakness	Epidural abscess, sacral osteomyelitis, arachnoiditis	Culture from evacuation of epidural abscess	Amikacin, linezolid, meropenem, azithromycin, tigecycline	Resolution
5	39	M	Sarcoidosis, heterozygous IRF8 variant	253	*Mycobacterium avium complex* (SGM)	Dysarthria, left hemiparesis, right cerebellar signs	Multiple enhancing intracranial mass (left frontal, right parietal, right cerebellar)	Tissue culture from resection of intracranial mass	Azithromycin, ethambutol, rifampin	Resolution
6	31	F	Anti-IFN-gamma autoantibodies	322	*Mycobacterium avium complex* (SGM)	Headaches, altered mental status	Extension of epidural abscess into right frontal parenchyma	Tissue culture from skull biopsy	Azithromycin, ethambutol, linezolid, moxifloxacin, clofazimine + Rituximab, bortezomib, daratumumab	Resolution

AR, auramine-rhodamine; CSF, cerebrospinal fluid; CN, cranial nerve; F, female; FLAIR, fluid-attenuated inversion recovery; IFN, interferon; IRF8, Interferon Regulatory Factor 8; M, male; N/A, not applicable; NEMO, Nuclear factor-kappa B Essential Modulator; PCR, polymerase chain reaction; PRES, posterior reversible encephalopathy syndrome; SGM, slow growing mycobacteria; RGM, rapidly growing mycobacteria.

## 3 Results

A total of 6 NIH cases were identified, which are summarized in Table 1. Our search using the microbiological query yielded a total of 5 cases, of which 3 were included (cases 1–3) and 2 were excluded as they were determined clinically to be due to specimen contamination. The search of consult records yielded 7 potential cases, only 3 of which were included upon review (cases 4–6) and 4 were excluded (2 cases of extra-neural infection without neurological symptoms, 1 case of *Mycobacterium tuberculosis* complex, and 1 pediatric case). Cases 4–6 were not identified by the microbiological query as the microbiological diagnosis was established outside NIH.

### 3.1 Clinical narratives of cases

#### 3.1.1 Case 1

**Figure 1:**
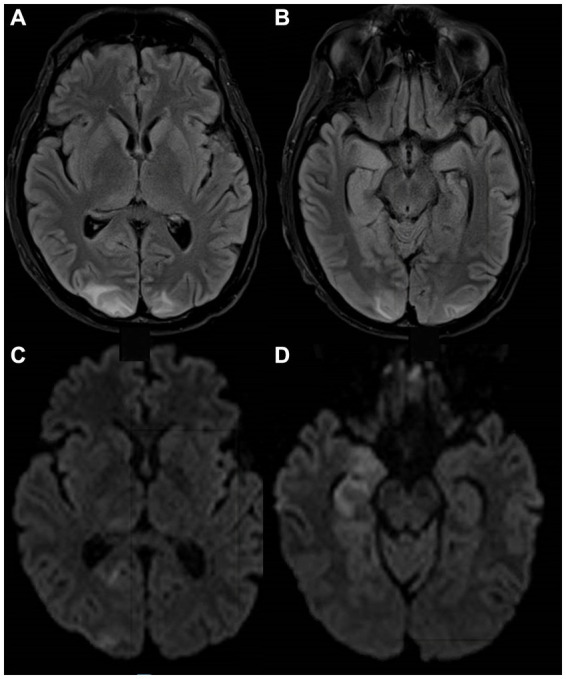
MRI images from a patient with NEMO deficiency and MAC infection. A 40-year-old man with NEMO deficiency and disseminated MAC infection. Images were obtained following a generalized seizure and show T2-FLAIR hyperintense signal in the bilateral occipital lobes **(A)** and the hippocampi **(B)**, initially considered to be posterior reversible encephalopathy syndrome vs. a post-ictal etiology due to the atypical distribution and restriction of some lesions on DWI **(C,D)**, but a post-mortem analysis of the brain showed microglial activation and nodules in the hippocampi, with tissue culture positive for MAC. DWI, diffusion-weighted imaging; FLAIR, fluid-attenuated inversion recovery; MAC, *Mycobacterium avium complex*; NEMO, Nuclear factor-kappa B Essential Modulator; PRES, posterior reversible encephalopathy syndrome.

A 33-year-old man presented with diffuse lymphadenopathy and multiple skin lesions from which tissue cultures were positive for MAC, and a diagnosis of disseminated MAC infection involving the skin, lymph nodes, and bone marrow was made. He was seen at the NIH and was diagnosed with immunodeficiency secondary to nuclear factor-kappa B essential modulator (NEMO) deficiency (*IKBKG* c.-16 + 1G > C leading to disruption of the intron-1 splice site). Despite long-term anti-mycobacterial treatments, with adjuvant interferon-gamma and interleukin-2, microbial burden fluctuated, with multiple episodes of clinical and laboratory worsening, including the development of lung, liver, and spleen involvement. At 40 years of age, he developed additional abdominal lesions, which were thought to be consistent with his known mycobacterial disease, and it was decided to surgically remove these to promote response to treatment. Following abdominal surgery, he experienced an acute change in mental status with visual hallucinations, and an MRI of the brain with gadolinium was normal, although no T2 fluid-attenuated inversion recovery (T2-FLAIR) sequence was acquired. Lumbar puncture showed mildly elevated opening pressure (25 cm H_2_O), but CSF was normal with a negative acid-fast bacilli (AFB) smear, fungal culture, and negative PCR for viral pathogens. A later generalized tonic–clonic seizure was treated with levetiracetam and lacosamide and required intubation. Three days later, a brain MRI demonstrated lesions in bilateral occipito-parietal and cerebellar regions with a high diffusion signal suggesting vasogenic edema and a few superimposed small foci of restricted diffusion in the cerebellum thought to be posterior reversible encephalopathy syndrome (PRES). In addition, there was diffuse hyperintensity with restricted diffusion involving the right pulvinar nucleus, right hippocampus, and amygdala, possibly suggesting seizure-induced changes (Figures 1A–D). He gradually improved and was extubated 4 days later. Two subsequent unenhanced MRI scans showed improvement in hyperintensities and diffusion restriction findings. The patient died a few weeks later of abdominal surgical complications. At autopsy, brain sections showed diffuse microglial activation with microglial nodule formation, parenchymal vacuolization, gliosis, and clusters of histiocytes. These changes were particularly prominent in the hippocampus, with evidence of neuronal dropout. Cultures were positive for MAC from multiple organs, including the brain (positive at 3 days and 20 h). CSF culture from earlier in the disease course was also positive for MAC at 28 days and 5 h. This case has been previously reported as Patient B.II.3 in a brief report on NEMO deficiency leading to chronic disseminated mycobacterial infections (9).

#### 3.1.2 Case 2

**Figure 2:**
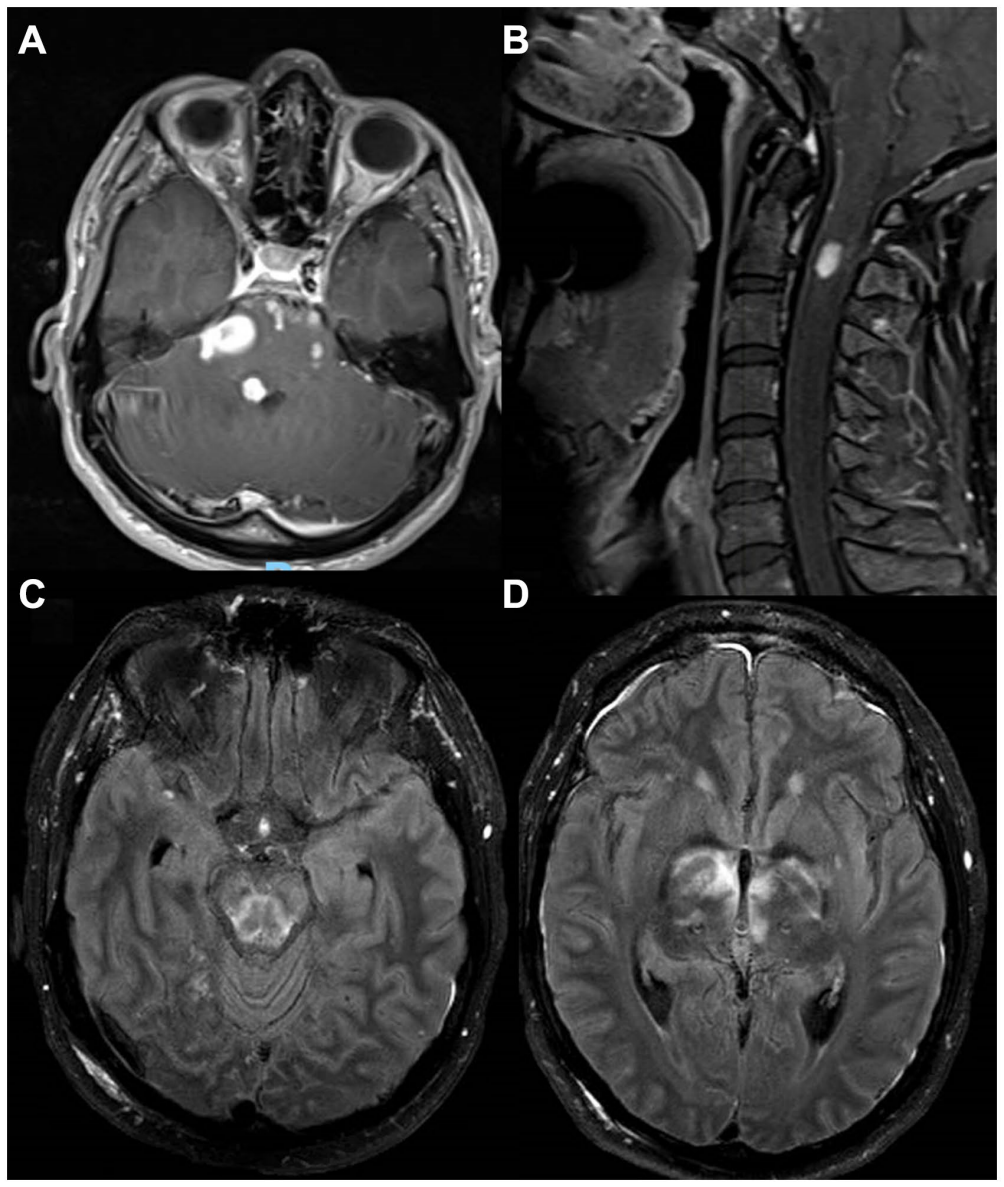
MRI images from a patient with NEMO deficiency and *Mycobacterium haemophilum* infection. A 28-year-old man with NEMO deficiency and *Mycobacterium haemophilum* infection with multiple enhancing lesions and associated edema on postcontrast T1 images from the brainstem **(A)** and upper cervical cord **(B)**. Despite treatment, he had persistent rhomboencephalitis on postcontrast T2-FLAIR 5 months later **(C,D)**, eventually leading to his death. FLAIR, fluid-attenuated inversion recovery; NEMO, Nuclear factor-kappa B Essential Modulator.

A 28-year-old man with a past medical history of recurrent childhood sinusitis presented with a 9-month history of elbow and facial rash, and 2 weeks of headaches and vomiting, right facial numbness, and gait instability. MRI of the brain showed multiple nodular and ring-enhancing lesions of the brainstem (Figure 2A) and upper cervical cord (Figure 2B) with associated edema, in addition to one focus of enhancement in the left frontal lobe. Biopsies of the skin and frontal lobe lesions showed necrotizing granulomatous inflammation with AFB without identification of a specific mycobacterial species. He was started on an empiric antituberculosis regimen, including isoniazid, rifampin, pyrazinamide, and ethambutol. However, 4 months later, while he was recovering from a hospital-acquired *Pseudomonas aeruginosa* pneumonia, molecular results from the biopsy identified *Mycobacterium haemophilum,* and he was switched to rifabutin, ciprofloxacin, and clarithromycin, but a month later was admitted again due to vomiting and ataxia. Brain MRI showed worsening edema, and he was treated with dexamethasone. Over the following months, symptoms recurred whenever steroids were tapered. He was subsequently treated with clofazimine, amikacin, and linezolid, and ciprofloxacin was switched to moxifloxacin. Two months later, he was first admitted to the NIH. Blood tests revealed low CD4+ lymphocyte count (203 cells/mm^3^), and he underwent whole genome sequencing wherein a pathogenic hemizygous mutation in NEMO was identified (*IKBKG* c.-16 + 1G > C leading to disruption of the intron-1 splice site). On examination, he had decreased facial sensation on the right, subtle impaired dexterity in his left limbs, with hyperreflexia and an extensor plantar response on the left. He was given a multi-antimycobaterial regimen including clarithromycin, moxifloxacin, rifabutin, pyridoxine, bedaquiline, clofazamine, dexamethasone, and linezolid. A month later, a brain MRI showed improvement in hyperintensities, swelling, and enhancement in the brainstem, with minimal punctate residual enhancement at the pontomedullary junction and medulla. CSF showed no white blood cells (WBCs) with elevated protein (62 mg/dL) and normal glucose (66 mg/dL). At this point, IFN-gamma was added to his regimen. Over the following months, his hospital course was complicated by multiple ICU admissions for hypoxemic and hypercapnic respiratory failure, being attributed to rhombencephalitis, with exacerbation from sedating medications used intermittently for agitation associated with respiratory distress. He had progressive quadriparesis with preserved ocular movements. Brain MRI showed extensive brainstem signal abnormality (Figure 2C) and progressive bi-thalamic and right basal ganglia signal changes on FLAIR (Figure 2D) with subtle enhancement. Following a 5-month hospitalization, he developed profound hypotension with tachycardia and died. At autopsy, gross examination of coronal sections revealed abnormal brainstem with midbrain and pons showing areas of softening and discoloration. Microscopically, the sections showed mycobacterial encephalitis with infarcts and numerous perivascular and parenchymal histiocytic aggregates loaded with mycobacteria. The hypothalamus, basal ganglia, brainstem, and cerebellum were involved. *Mycobacterium haemophilum* DNA was detected in the brainstem using a 16S ribosomal RNA gene primer set.

#### 3.1.3 Case 3

**Figure 3:**
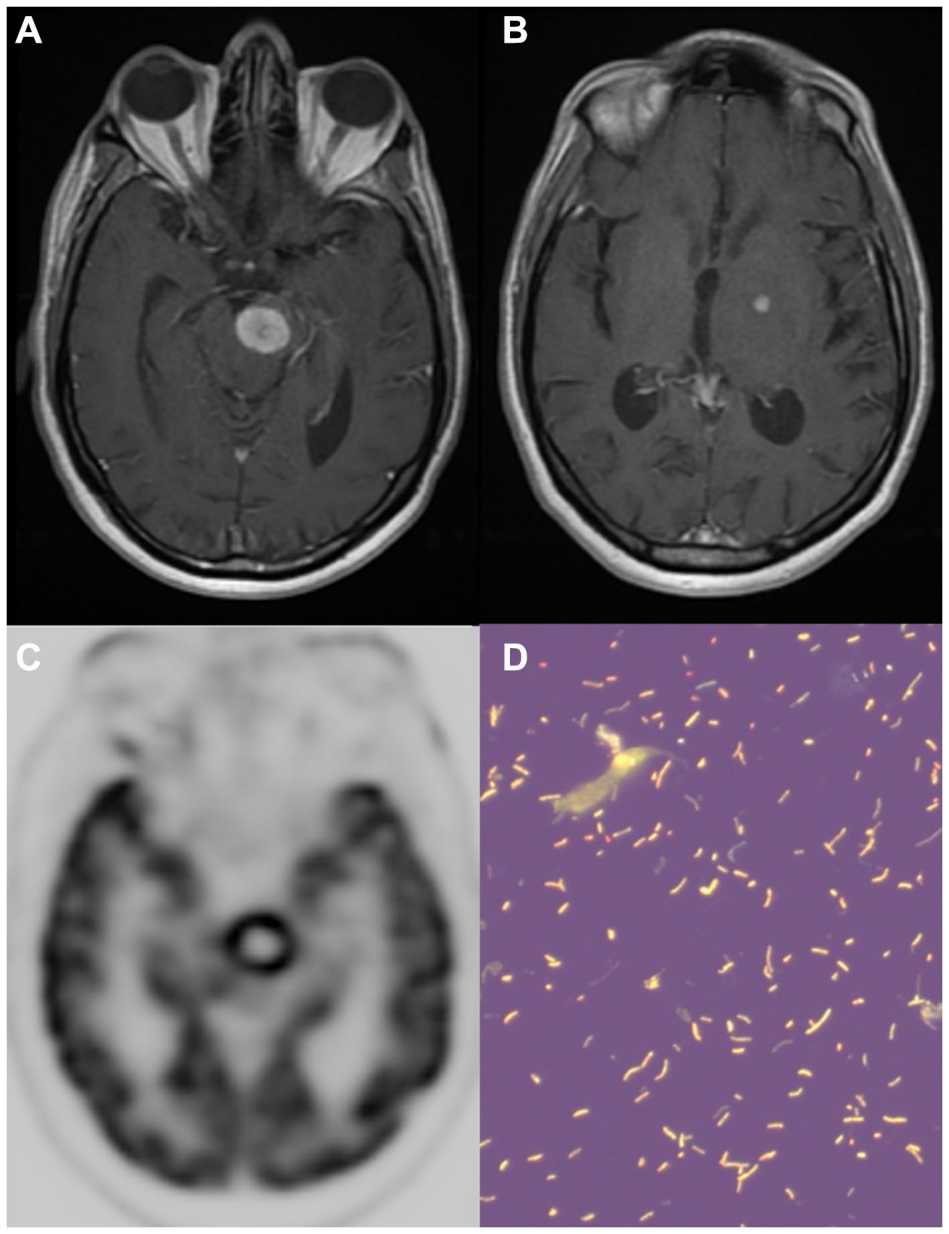
A 58-year-old man with untreated HIV and *Mycobaterium avium subspecies paratuberculosis* infection. A large enhancing lesion in the left midbrain [**(A)** – postcontrast T1], with a center of hypointensity on susceptibility-weighted imaging (not shown). In addition, multiple small enhancing lesions were noted in the left frontal lobe (not shown) and deep gray matter **(B)**. On PET-FDG **(C)**, the lesion had high FDG uptake in the periphery (maximal standardized uptake value = 12.8) with an area of decreased uptake centrally that corresponds to the area of hemorrhagic transformation. Tissue from a stereotactic left midbrain biopsy was positive on the auramine-rhodamine stain **(D)**. PET-FDG, positron emission tomography with fluorodeoxyglucose.

A 58-year-old man with a past medical history of untreated HIV infection since the age of 32 years presented with 3 months of progressive right-sided weakness and numbness, visual disturbance, and recurrent falls. His examination showed mild impairment of attention, mild dysarthria, right central facial palsy, left oculomotor nerve palsy, and mild right hemiparesis. CD4+ cell count was 110 cells/mm^3^, and HIV RNA viral load was 79,400 copies/mL. Brain MRI showed a large enhancing lesion in the left midbrain (Figure 3A) with central hypointensity on susceptibility-weighted imaging. There was edema associated with the lesion but no restricted diffusion. Additionally, other small enhancing lesions were present in the left frontal lobe and deep gray matter (Figure 3B). Loss of volume of the cerebral hemispheres and confluent white matter signal abnormalities were also present, consistent with HIV encephalopathy. Serum *Toxoplasma gondii* IgG and IgM were negative, and chest-abdomen-pelvis CT scan was normal. CSF showed 9 WBCs per mm^3^, with elevated protein (85 mg/dL) and normal glucose (76 mg/dL). CSF was positive for EBV-DNA (491 copies/mL), with a negative PCR for *Toxoplasma gondii*. The main differential diagnosis at this point was primary CNS lymphoma vs. CNS toxoplasmosis. Due to the high-risk location of the large lesion, he was transferred to the NIH for further management. At the NIH, he was first treated empirically for toxoplasmosis for 7 days. A whole body FDG-PET scan showed evidence of esophagitis and dental abscesses with no evidence of systemic lymphoma. The left midbrain lesion showed high FDG uptake in the periphery (maximal standardized uptake value = 12.8, Figure 3C) with an area of decreased uptake centrally that corresponded to the area of hemorrhagic transformation seen on MRI. Eventually, a stereotactic left midbrain biopsy was performed, and histologic sections showed a fibrinous matrix containing numerous neutrophils and few admixed mononuclear cells, with stains positive for numerous AFB and a positive auramine-rhodamine stain (Figure 3D). Pan-Mycobaterium SecA1 real-time PCR assay identified the pathogen as *Mycobaterium avium* subspecies *paratuberculosis*. He was treated with rifabutin, ethambutol, azithromycin, and moxifloxacin, in addition to a 4-week intravenous course of meropenem to treat dental abscesses and the possibility of polymicrobial brain abscesses. Oral steroids were also given (dexamethasone and later prednisone). Brain MRI 1 week after initiation of anti-mycobacterial regimen showed interval growth of enhancing lesions, but a scan 2 weeks later showed mildly decreased size of the left midbrain lesion (not shown). At this point, antiretroviral treatment was initiated, and he was also treated for biopsy-proven CMV esophagitis. His post-antiretroviral treatment course was initially complicated by clinical and radiological features of immune reconstitution inflammatory syndrome, requiring an intermittent increase of steroid dose, yet on subsequent follow-up, right hemiparesis gradually improved, which correlated with a gradual decline in size and associated edema of the brain lesions on MRI. The patient fully recovered clinically with only residual radiological findings, including a small hemorrhagic ring-enhancing lesion in the left midbrain and subtle residual foci of enhancement in the left frontal lobe.

#### 3.1.4 Case 4

**Figure 4:**
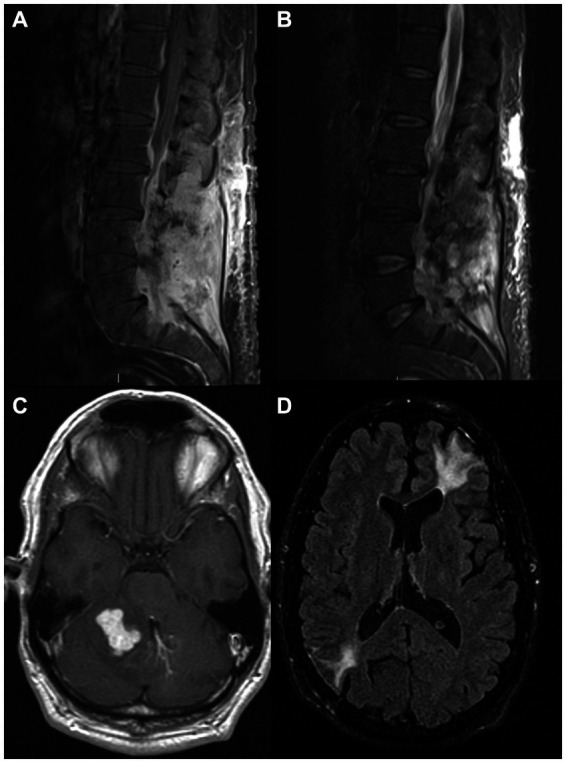
MRI images from two patients with neurological manifestations of nontuberculous mycobacteria. MRI images from a 56-year-old man with *Mycobacterium abscessus* isolated from a lumbosacral epidural abscess with sacral osteomyelitis and arachnoiditis [**(A)** – postcontrast T1, **(B)**– STIR], and a 39-year-old man with sarcoidosis and IRF-8 variant with a cerebellar enhancing lesion at presentation **(C)** and an encephalomalacia years after resection of left frontal and right parietal lesions due to *Mycobacterium avium* complex **(D)**. IRF8, Interferon Regulatory Factor 8; STIR, short tau inversion recovery.

A 56-year-old man with a history of hypertension, chronic lower back pain, and multiple abdominal surgeries due to a gunshot wound around the age of 20 years presented to the emergency room with worsening lower back pain and right leg pain 8 weeks after he received a corticosteroid epidural injection for his chronic pain. Initially, a lumbar MRI showed only focal arachnoiditis, and he was treated with oral corticosteroids, but a week later, he had a fever and worsening pain, and a repeat MRI showed an epidural phlegmon with a focal abscess which displaced the thecal sac to the left from L4-L5 to mid-S1. He was treated empirically with IV antibiotics and underwent L5-S1 laminectomy and evacuation of the epidural abscess without complications. Pathology showed fragments of granulation tissue with focal giant cell reaction and was AFB+, while tissue culture was positive for *Mycobacterium abscessus* sensitive to amikacin. HIV testing was negative. Three weeks after antibiotic regimen initiation (unclear documentation), the MRI revealed continued enhancement with no fluid collections. A repeat MRI 3 weeks later showed a phlegmon extending from L2-L5 with fluid pockets impinging the dural sac and causing severe canal stenosis, in addition to focal osteomyelitis in the right sacrum. There was also an extension of abnormal enhancement along the right-sided sacral nerve roots in the right presacral region. A repeat laminectomy and drainage procedure was performed, and he was given amikacin, linezolid, meropenem, and azithromycin. He was then transferred to the NIH. Upon arrival, his examination showed minimal weakness in the right leg. Lumbar MRI showed stable epidural abscess and sacral osteomyelitis (Figures 4A,B), with some resolution of the fluid in the post-laminectomy space. In addition, thickening and enhancement of the lumbar nerve roots were noted, consistent with arachnoiditis. Tigecycline was added to the antimicrobial regimen, and eventually, amikacin was stopped because of concern for ototoxicity. Serial MRI scans showed a decrease in the amount of post-operative fluid collection, and additional neurosurgical intervention was determined unnecessary. He continued this antimicrobial regimen for 7 months, then continued linezolid and azithromycin for an additional 5 months. One year after completing treatment, he had no symptoms and no signs of recurrence.

#### 3.1.5 Case 5

A 39-year-old man with sarcoidosis diagnosed at 35 years by lung biopsy, previously treated with prednisone for 2 years, had a motor vehicle accident due to a possible seizure. Head CT demonstrated multiple enhancing intracranial masses. A biopsy of a left frontal mass revealed MAC by culture, for which monotherapy with IV amikacin was initiated. A few months later, a right parietal mass was resected with cultures again confirming MAC, and he was started on clarithromycin, ethambutol, and rifampin. At this point, he was referred to the NIH. Neurologic examination showed mild dysarthria, mild left hemiparesis, and right cerebellar signs. Serial brain MRI scans showed an enlarging right cerebellar lesion (Figure 4C) without hydrocephalus. Treatment with gamma-interferon was considered, but there was concern that this might increase cerebral edema, and, therefore, he underwent resection of his cerebellar lesion. MAC was again identified in the tissue. The patient improved clinically over the next few months, and a follow-up brain MRI showed post-operative changes (Figure 4D) with a stable appearance of a left posterior parasagittal enhancing nodule, which was not resected. After completing his initial course of antimicrobial treatment, he remained on secondary prophylaxis with azithromycin. He was found to have a heterozygous Interferon Regulatory Factor 8 (IRF8) variant (c.536C > T, p.ALA179VAL) of uncertain significance. The patient had no recurrence of his mycobacterial disease on annual follow-ups, although he did have several exacerbations of sarcoidosis. Clinical information regarding this case has been previously reported before the identification of the additional underlying genetic association (10).

#### 3.1.6 Case 6

**Figure 5:**
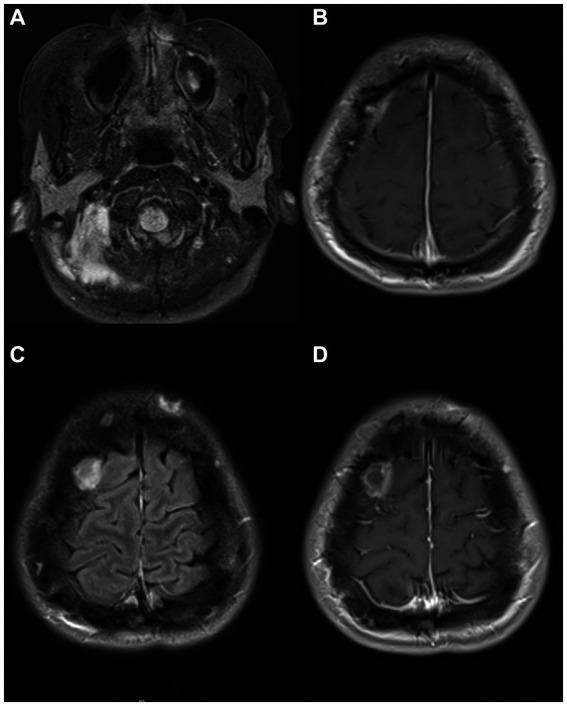
MRI images from a 31-year-old woman with anti-IFN-γ autoantibodies and *Mycobacterium avium* infection. Brain MRI images showing a left post-auricular lesion with erosion through the bone [**(A)** – postcontrast FLAIR] and a right frontal epidural collection [**(B)** – postcontrast T1], later also extending into right frontal parenchyma [**(C)** – postcontrast FLAIR, **(D)**– postcontrast T1]. FLAIR, fluid-attenuated inversion recovery; IFN, interferon.

A 31-year-old woman originally from the Philippines, previously healthy, presented with recurrent fevers, headaches, and painful post-auricular nodules. She was empirically treated with azithromycin for possible sinusitis without improvement. Brain MRI showed a 3 cm X 2 cm left post-auricular lesion with erosion through the bone (Figure 5A), as well as a right frontal extra-axial convexity lesion (Figure 5B). Sputum and aspirate from a skull biopsy were AFB positive and grew MAC. She was initially treated with amikacin, azithromycin, and ethambutol (had an anaphylactic reaction to rifampin) and underwent drainage of left occipital and left paraspinal abscesses. Further investigations showed that she had anti-IFN-gamma autoantibodies and, therefore, rituximab 1 g once monthly was started. However, the patient’s headaches persisted, and she developed blurry vision with bilateral optic disc edema. A lumbar puncture revealed an opening pressure of 31 cm H_2_O with lymphocytic meningitis, at which point moxifloxacin and linezolid were added. Her headaches persisted, so a lumbar drain was placed for 1 week. She was then referred to the NIH, where bedaquiline and meropenem were added to her regimen. Following 5 additional months of rituximab and an antimicrobial regimen (initially including azithromycin, ethambutol, linezolid, moxifloxacin, and clofazimine), she still had evidence of clinical and radiological progression. Tidezolid and meropenem were added, and to decrease autoantibody production, bortezomib (a small-molecule proteasome inhibitor used in the treatment of multiple myeloma) was initiated at 1.3 mg/m^2^ subcutaneous injection following the standard regimen but was discontinued after 3 months due to hepatotoxicity. Despite the continuation of rituximab to maintain CD20 numbers undetectable, clinical and radiographic disease progressed. To further reduce autoantibody production, she was started on daratumumab, an anti-CD38 monoclonal antibody (16 mg/kg intravenously weekly) also used in the treatment of multiple myeloma. She initially had worsening headaches and altered mental status, with a brain MRI showing an extension of epidural abscess into the right frontal parenchyma (Figures 5C,D). A lumbar puncture showed an opening pressure of 33 cm H_2_O and pleocytosis (59 WBCs per mm^3^), mildly increased protein (61 mg/dL), and low glucose (47 mg/dL, CSF to serum ratio of 0.4). She underwent abscess evacuation without complications. Following this, she continued treatment with daratumumab along with IVIG for secondary hypogammaglobulinemia, with clinical and radiographic improvement, reduced pain, and disappearance of multiple soft tissue lesions. The successful use of daratumumab in this case has been previously reported (11).

### 3.2 Review of literature

Our search yielded 2,588 publications, of which 81 reports met the inclusion criteria. The exclusion flowchart is presented in Supplementary Figure S1. These 81 reports encompassed a description of 125 cases of adult NTM infection with neurological manifestations (3–8, 12–86). They originated most commonly from North America (39, 48%), with the rest originating from Europe (20), Asia (16), Latin America (3), Australia (2), and Africa (1). Sixty-two percent (77 of 125) of the cases occurred in men, and the median patient age was 39 years (range 19–97 years). Of all cases, 85 (68%) were caused by SGM, most commonly MAC (*n* = 61), while 38 cases (30%) were caused by RGM, most frequently *Mycobacterium abscessus* (*n* = 21). In two additional cases, the mycobacterial species were not specified. Interestingly, no definite cause for susceptibility to infection was identified in 26 cases (21%), although the rigor of the search was not provided.

Table 2 presents the clinical and radiological features of the cases found in the literature, grouped into SGM vs. RGM. Overall, within SGM cases (*n* = 85), the most common underlying disease was HIV infection (*n* = 55, 65%), with a median CD4+ cell count of 29 cells/mm^3^ (range 2–404). In RGM cases, the most common underlying condition was a recent neurosurgical intervention or the presence of an implant (21 of 38 cases, 55%). In both groups, the most common presenting symptoms were headaches and encephalopathy. However, seizures were significantly more common in the SGM group (29% vs. 8%, *p* = 0.01), correlating with the higher frequency of distinct focal lesions (54% vs. 18%, *p* = 0.001). Headaches were more common in the RGM group (84% vs. 55%, *p* = 0.002), as were meningeal signs (24% vs. 7%, *p* = 0.007). CSF analysis was the most common method of diagnosis in both groups, but tissue-based diagnosis was used in a larger proportion of the SGM group (39% vs. 13%, *p* = 0.04), and tissue AFB-stains were commonly positive in this group (68%). AFB-stain positivity rates of CSF were low in both groups but slightly higher in RGM cases (33% vs. 14%, *p* = 0.04). Survival rates were similar in both groups, 55% in the RGM group and 48% in the SGM group. Within pathogen subgroups, a high mortality rate was noted in the *Mycobacterium haemophilum* cases (83%), but this was not statistically significant due to the small number of cases. In the final multiple regression model, factors associated with survival, after adjusting for age and sex, were a solitary CNS lesion [odds ratio (OR) 5.9, 95% confidence interval (CI) 1.5–20.9, *p* = 0.01], and a diagnosis made by CSF sampling only (OR 9.9, 95%CI 1.6–194, *p* = 0.04). In this model, age was not associated with survival rates, but female cases had significantly higher rates of survival (OR 3.6, 95%CI 1.5–9, *p* = 0.007).

**Table 2:** Summary of clinical, microbiological, and radiological characteristics of previously reported cases of non-tuberculosis mycobacteria with neurological manifestations.

		Total cases (*n* = 125)	Slow-growing mycobacteria					Rapidly growing mycobacteria			
			Total (*n* = 85)	MAC (*n* = 61)	*M. kansasii* (*n* = 7)	*M. haemophilum* (*n* = 6)	Other (*n* = 11)	Total (*n* = 38)	*M. abscessus* (*n* = 21)	*M. fortuitum* (*n* = 9)	Other (*n* = 8)
Age, years, mean ± SD		43 ± 15	43 ± 14	41 ± 14	42 ± 17	49 ± 11	47 ± 16	45 ± 18	48 ± 18	47 ± 23	37 ± 11
Sex, male, *n* (%)		77 (62)	59 (69)	42 (69)	4 (57)	5 (83)	8 (73)	16 (42)*	9 (43)	5 (56)	2 (25)
Underlying condition	HIV	59 (47)	55 (65)	41 (67)	5 (71)	5 (83)	4 (36)	2 (5)*	0 (0)	1 (11)	1 (12.5)
	Neurosurgery/implant	24 (19)	3 (4)	3 (5)	0 (0)	0 (0)	0 (0)	21 (55)*	14 (67)	5 (56)	2 (25)
	Sarcoidosis	4 (3)	4 (5)	4 (7)	0 (0)	0 (0)	0 (0)	0 (0)	0 (0)	0 (0)	0 (0)
	Hematologic malignancy	4 (3)	4 (5)	2 (3)	0 (0)	0 (0)	2 (18)	0 (0)	0 (0)	0 (0)	1 (12.5)
	Immunosuppressive treatment	6 (5)	4 (5)	3 (5)	0 (0)	1 (17)	0 (0)	2 (5)	1 (5)	0 (0)	0 (0)
	Genetic	2 (2)	1 (1)	1 (2)	0 (0)	0 (0)	0 (0)	1 (3)	1 (5)	0 (0)	0 (0)
	Non identified	26 (21)	14 (16)	7 (11)	2 (29)	0 (0)	5 (45)**	12 (32)	0 (0)	3 (33)	4 (50)
Neurological manifestations	Headache	79 (63)	47 (55)	37 (61)	4 (57)	0 (0)**	6 (54)	32 (84)*	19 (90)	6 (67)	7 (87.5)
	Encephalopathy	61 (49)	38 (45)	28 (46)	3 (43)	2 (33)	5 (45)	22 (58)	16 (76)***	3 (33)	3 (37.5)
	Focal deficit	41 (33)	31 (36)	21 (34)	2 (29)	3 (50)	5 (45)	9 (24)	2 (10)	2 (22)	5 (62.5)***
	Seizure	28 (22)	25 (29)	22 (36)	1 (14)	1 (17)	1 (9)	3 (8)*	1 (5)	0 (0)	2 (25)
	Meningeal signs	16 (13)	6 (7)	6 (10)	0 (0)	0 (0)	0 (0)	10 (26)*	4 (19)	5 (56)***	1 (12.5)
Imaging features	Focal lesions	55 (44)	46 (54)	26 (43)	6 (86)**	6 (100)**	8 (73)	7 (18)*	3 (14)	2 (22)	2 (25)
	Single (% of focal)	22 (40)	20 (43)	12 (46)	3 (50)	1 (17)	4 (50)	2 (29)	1 (33)	1 (50)	0 (0)
	Multiple (% of focal)	33 (60)	26 (57)	14 (54)	3 (50)	5 (83)	4 (50)	5 (71)	2 (67)	1 (50)	2 (100)
	Hydrocephalus	5 (4)	4 (5)	1 (2)	0 (0)	0 (0)	3 (27)**	1 (2)	1 (5)	0 (0)	0 (0)
Other organ involvement	Disseminated	22 (18)	17 (20)	12 (20)	2 (29)	1 (17)	2 (18)	5 (13)	3 (14)	1 (11)	1 (12.5)
	Pulmonary	8 (6)	4 (5)	1 (2)	3 (43)	0 (0)	0 (0)	3 (8)	1 (5)	2 (22)	0 (0)
	Other^a^	5 (4)	2 (2)	0 (0)	0 (0)	1 (17)	1 (9)	3 (8)	0 (0)	0 (0)	2 (25)
Microbiological diagnosis	CSF	65 (52)	42 (49)	35 (57)	0 (0)	0 (0)	7 (64)	21 (55)	8 (38)***	7 (78)	6 (75)
	Culture (% of CSF)	56 (86)	37 (88)	33 (94)	0 (0)	0 (0)	4 (57)**	17 (81)	8 (100)	5 (71)	4 (67)
	Molecular^b^ (% of CSF)	9 (14)	5 (12)	2 (6)	0 (0)	0 (0)	3 (43)**	4 (19)	0 (0)	2 (29)	2 (33)
	AFB-stain pos rate (%)	9 (14)	2 (5)	1 (3)	0 (0)	0 (0)	1 (14)	7 (33)*	6 (75)***	1 (14)	0 (0)
	CNS tissue	38 (30)	33 (39)	19 (31)	4 (57)	6 (100)	4 (36)	5 (13)*	2 (10)	1 (11)	2 (25)
	Culture (% of tissue)	25 (66)	23 (70)	13 (68)	4 (57)	3 (50)	3 (75)	2 (40)	1 (50)	0 (0)	1 (50)
	Molecular^b^ (% of CSF)	13 (34)	10 (30)	6 (32)	0 (0)	3 (50)	1 (25)	3 (60)	1 (50)	1 (100)	1 (50)
	AFB-stain pos rate (%)	26 (68)	25 (76)	15 (79)	3 (75)	5 (83)	2 (50)	1 (20)	1 (50)	0 (0)	0 (0)
	Other tissue culture^c^	9 (7)	8 (9)	5 (8)	3 (43)**	0 (0)	0 (0)	1 (3)	0 (0)	1 (11)	0 (0)
	Unspecified CNS source	13 (10)	2 (2)	2 (3)	0 (0)	0 (0)	0 (0)	11 (29)	11 (52)	0 (0)	0 (0)
Antimicrobial regimen	Fluoroquinolone	31 (25)	13 (15)	7 (11)	1 (14)	2 (33)	3 (27)	17 (45)*	11 (52)	4 (44)	2 (25)
	Ciprofloxacin	13 (10)	9 (10)	5 (8)	1 (14)	1 (17)	2 (18)	4 (11)	1 (5)	3 (33)	0 (0)
	Levofloxacin	7 (6)	1 (1)	0 (0)	0 (0)	1 (17)	0 (0)	6 (16)*	4 (19)	1 (11)	1 (12.5)
	Moxifloxacin	10 (8)	2 (2)	1 (2)	0 (0)	0 (0)	1 (9)	7 (18)*	6 (29)	0 (0)	1 (12.5)
	Macrolide	53 (42)	26 (31)	18 (30)	0 (0)	3 (50)	5 (45)	26 (68)*	19 (90)***	3 (33)	4 (50)
	Azithromycin	11 (9)	7 (8)	5 (8)	0 (0)	2 (33)	0 (0)	3 (8)	2 (10)	3 (33)	1 (12.5)
	Clarithromycin	42 (34)	19 (22)	13 (21)	0 (0)	1 (17)	5 (45)	23 (61)*	17 (81)***	0 (0)	3 (37.5)
	Rifamycins	39 (31)	35 (41)	19 (31)	6 (86)**	3 (50)	7 (64)**	3 (8)*	0 (0)	1 (11)	2 (25)
	Rifampicin	27 (22)	24 (28)	12 (20)	5 (71)**	2 (33)	5 (45)	3 (8)	0 (0)	1 (11)	2 (25)
	Rifabutin	12 (10)	11 (13)	7 (11)	1 (14)	1 (17)	2 (18)	0 (0)	0 (0)	0 (0)	0 (0)
	Ethambutol	38 (30)	36 (42)	20 (32)	6 (86)**	2 (33)	8 (73)**	1 (3)*	0 (0)	0 (0)	1 (12.5)
	Isoniazid	16 (13)	15 (18)	3 (5)	6 (86)**	1 (17)	5 (45)**	1 (3)*	0 (0)	0 (0)	1 (12.5)
	Other										
	TMP/SMX	6 (5)	1 (1)	1 (2)	0 (0)	0 (0)	0 (0)	5 (13)*	2 (10)	1 (11)	2 (25)
	Carbapenem	10 (8)	2 (2)	1 (2)	0 (0)	1 (17)	0 (0)	8 (21)*	7 (33)	1 (11)	0 (0)
	Amikacin	25 (20)	6 (7)	5 (8)	0 (0)	0 (0)	1 (9)	19 (50)*	13 (62)	3 (33)	3 (37.5)
	Clofazimine	4 (3)	4 (5)	3 (5)	0 (0)	1 (17)	0 (0)	0 (0)	0 (0)	0 (0)	0 (0)
	Pyrazinamide	6 (5)	5 (6)	2 (3)	0 (0)	1 (17)	2 (18)	1 (3)	0 (0)	0 (0)	1 (12.5)
Clinical/radiological response to regimen	Complete resolution	32 (26)	21 (24)	15 (25)	2 (29)	1 (17)	3 (27)	11 (29)	1 (5)	5 (56)***	3 (37.5)
	Improvement	18 (14)	14 (16)	7 (11)	4 (57)**	0 (0)	3 (27)	3 (8)	3 (14)	1 (11)	1 (12.5)
	Worsening	31 (25)	18 (21)	8 (13)	1 (14)	4 (67)**	5 (45)	12 (32)	6 (29)	3 (33)	3 (37.5)
	Not specified	44 (35)	32 (38)	31 (51)	0 (0)	1 (17)	0 (0)	12 (32)	11 (52)	0 (0)	1 (12.5)
	Survival	62 (50)	41 (48)	32 (52)	3 (43)	1 (17)**	5 (45)	21 (55)	10 (48)	6 (67)	5 (62.5)

*Significantly different compared to slow-growing mycobacteria (*p* < 0.05). **Significantly different compared to other subgroups of slow-growing mycobacteria (*p* < 0.05). ***Significantly different compared to other subgroups of rapidly growing mycobacteria (*p* < 0.05). ^a^Other organ involvement – hepatic (*n* = 2), spleen (*n* = 1), and joints (*n* = 2). ^b^Molecular – PCR (polymerase chain reaction) or rRNAseq (ribosomal ribonucleic acid sequencing). ^c^Other tissue culture – sputum (*n* = 4), blood (*n* = 4), and bone (*n* = 1). AFB, acid fast bacillus; CNS, central nervous system; CSF, cerebrospinal fluid; M, mycobacterium; MAC, *Mycobacterium avium* complex; pos, positivity; SD, standard deviation; TMP/SMX, trimethoprim-sulfamethoxazole.

## 4 Discussion

Neurological manifestations of NTM are diverse and pose multiple challenges in diagnosis and treatment. Our report describes our experience with six cases and offers some important generalizable insights, especially when brought together with our detailed literature review of published case reports and small case series.

Based on the six cases reported and in comparison to the data from the literature review, several key points can be highlighted. First, our patients presented most commonly with focal deficits, consistent with the location of their lesions, as was commonly found in previous reports reviewed, especially in SGM cases (12–15). In our cohort, the diagnosis could only be made by tissue sampling in most cases, with CSF being insufficiently sensitive. This concept was partially supported by our review of the literature, showing that a tissue-based diagnosis was necessary in 30% of cases, more commonly in SGM cases, in which AFB-stain was also very rarely positive in the CSF (7%). This suggests that many NTM cases may be missed because of the lack of tissue sampling, allowing them to progress and be diagnosed in other ways. In addition to the diagnostic challenges, in one of our cases (case 1), the neurological involvement could not be definitively attributed to a mycobacterial infection of the CNS and was potentially attributed to PRES, although post-mortem analysis showed that the brain was diffusely infected, suggesting the CNS could potentially be sub-clinically involved in cases of NTM infection, especially in the setting of disseminated infection.

In terms of the underlying disease, all but one had an underlying immunodeficiency. Rare conditions were diagnosed in our cohort, including three patients with known genetic predispositions (two with mutations in NEMO and one in IRF8) and one with anti-interferon-gamma antibodies. These types of conditions have been very rarely reported in the literature on neurological NTM (only 2% of cases reported a genetic predisposition), while many cases (21%) remained without the identification of a predisposing factor. These data strongly reaffirm that for all cases of disseminated NTM, and neurologic disease in particular, diagnostic effort should include investigation for both primary and secondary immunodeficiency conditions, especially in cases without a history of neurosurgical intervention or implant. Specifically, our patients had predisposing factors involving the interferon-gamma pathway, which plays a key role in cellular immunity. It is essential to test for anti-interferon-gamma antibodies (87, 88) and for NEMO deficiency (9), which can be missed by whole exome sequencing.

Based on our literature review, some differences between neurological diseases caused by SGM and RGM were identified, especially in underlying conditions. RGM infections were associated mostly with neurosurgical procedures and implants, while SGM cases mostly occurred in patients with underlying immunodeficiencies, most commonly HIV. In addition, RGM cases primarily presented with a clinical syndrome consistent with meningitis or meningoencephalitis, while patients with SGM more commonly presented with seizures or focal deficits due to focal abscesses. This is in line with the well-recognized ability of RGM to form biofilms on prosthetic surfaces (89). While SGM, specifically MAC, can also form biofilms, it is more commonly a cause of a slowly developing pulmonary or disseminated disease in immunodeficient patients (90), which presumably leads to CNS entry through a hematogenous route. Most labs do not have the ability to easily identify all MAC subspecies (such as *paratuberculosis*), and determining whether there is a subspecies or virulence factor that promotes CNS infection will require future studies.

Treatment of NTM infections is complex and involves prolonged treatment durations and multi-drug regimens, including mostly fluoroquinolones, macrolides, rifamycins as well as ethambutol and isoniazid, and more recently novel drugs such as bedaquiline (91) While several guidelines have been published for the management of NTM disease (92, 93), none specifically address nervous system involvement. This was reflected in the variability of regimens used (Table 2) in the cases reported in the literature. In the literature review and in our cases, we could not find any association between survival and any specific antimicrobial regimen. Macrolides were the most commonly used antibiotics, both in RGM and SGM cases. This group of medications is known to have limited CSF concentrations in the absence of meningeal inflammation (94), and further investigation into its efficacy and pharmacodynamics in CNS infections is warranted. While our analysis for factors associated with survival is limited due to the retrospective nature of the study and heterogeneous treatments, we did find an indication that survival rates were higher in cases with a solitary CNS lesion, reflecting a possible better outcome attributed to contained lesions rather than a multifocal or diffuse disease. In addition, higher rates of survival were observed in cases in which the diagnosis was made by CSF sampling alone as opposed to cases requiring tissue sampling, which could also be mediated by underlying conditions and pathogen characteristics. Interestingly, male sex was associated with higher rates of mortality, which is consistent with previous reports regarding systemic NTM disease as well as tuberculosis (95). High mortality rates were noticed in cases of *Mycobacterium haemophilum*, including one of our cases. While these are small numbers, no such trend is reported in systemic cases (96), and further studies are required to assess if nervous system involvement of this pathogen carries a particular risk compared to other SGM.

There are limitations to our study, mainly the small sample size of our case series and the retrospective analysis of data extracted from previous records and literature reports. However, the combination of these two approaches provides unique observations into the characteristics of NTM infections with neurological manifestations, which could increase rates of diagnosis and improve the management of these patients. In conclusion, neurological manifestations can be the presenting feature of NTM without systemic involvement. It can appear in many forms, including meningitis and parenchymal lesions. It can present for the first time in adult life, even with predisposing genetic mutations and without a history of unusual infections. Genetic mutations, autoimmune conditions, viral infections, or drugs that downregulate the gamma interferon pathway put patients at increased risk for NTM. Tissue diagnosis is, in many cases, the only way to detect these pathogens, and when detected, aggressive treatment with a multi-drug regimen should be initiated, in addition to investigations for an underlying immunodeficiency if not previously known.

## Data availability statement

The raw data supporting the conclusions of this article will be made available by the authors, without undue reservation.

## Ethics statement

The studies involving humans were approved by Institutional Review Board at the NIH. The studies were conducted in accordance with the local legislation and institutional requirements. The participants provided their written informed consent to participate in this study.

## Author contributions

YM: Conceptualization, Formal analysis, Investigation, Writing – original draft, Writing – review & editing. AK: Data curation, Writing – original draft, Writing – review & editing. MM: Data curation, Writing – original draft, Writing – review & editing. DH: Data curation, Writing – original draft, Writing – review & editing. TW: Formal analysis, Writing – original draft, Writing – review & editing. JH: Writing – original draft, Writing – review & editing. IS: Data curation, Writing – original draft, Writing – review & editing. BS: Writing – original draft, Writing – review & editing. CZ: Data curation, Writing – original draft, Writing – review & editing. SH: Conceptualization, Data curation, Investigation, Supervision, Writing – original draft, Writing – review & editing. AN: Conceptualization, Data curation, Investigation, Methodology, Supervision, Writing – original draft, Writing – review & editing.
