# Supplementary figures and images for: Neurological manifestations of nontuberculous mycobacteria in adults: case series and review of the literature
Source: Front Neurol. 2024 Apr 26;15:1360128. doi: 10.3389/fneur.2024.1360128 (PMC11089811; doi:10.3389/fneur.2024.1360128)

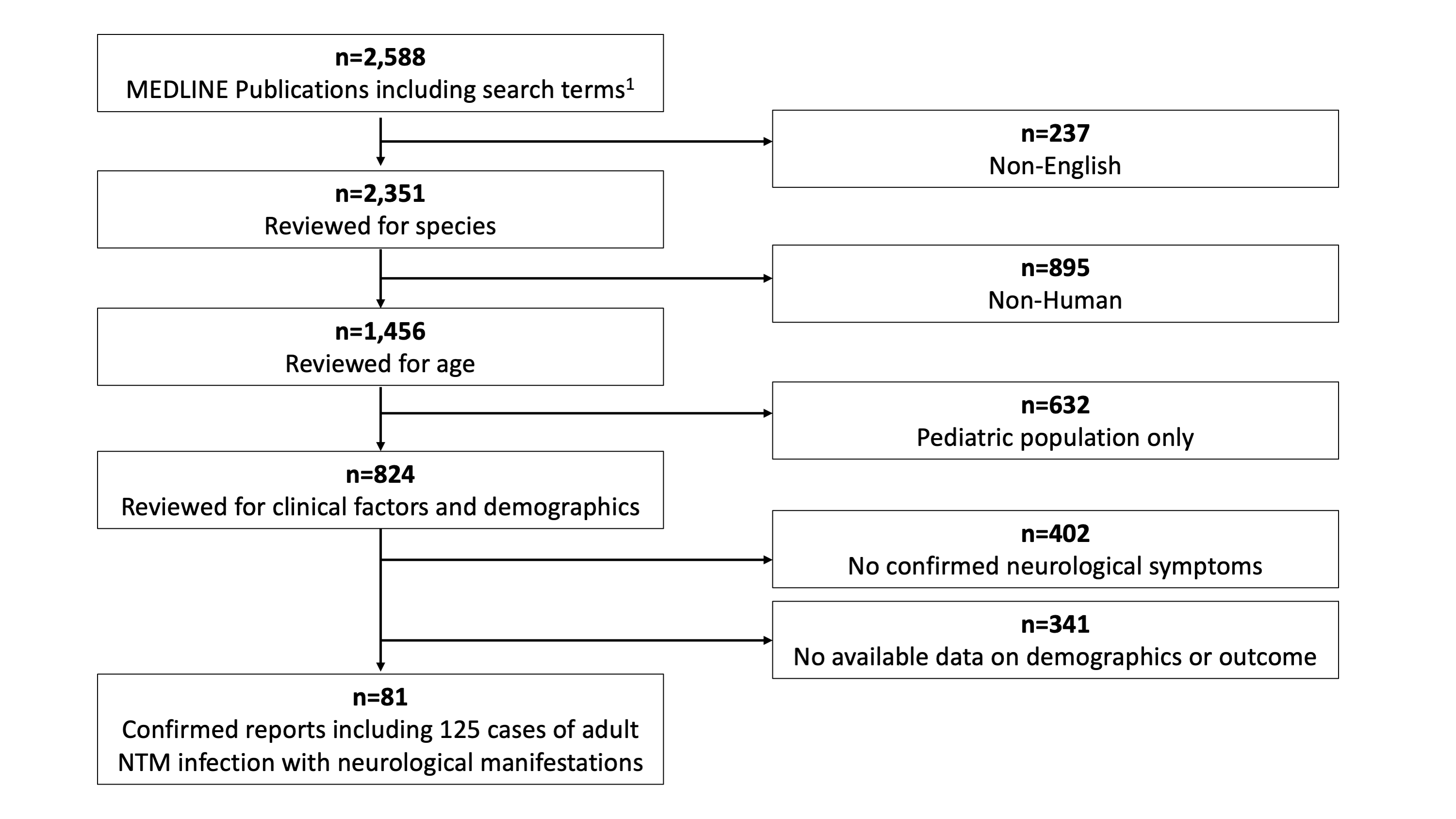

Supplement: Supplementary file 1 [file Image_1.TIFF]
